# ﻿Nomenclatural notes of *Frullaniaauriculata* (Frullaniaceae) and lectotypification of *Porellatakakii* (Porellaceae)

**DOI:** 10.3897/phytokeys.241.123013

**Published:** 2024-05-02

**Authors:** Tian-Xiong Zheng

**Affiliations:** 1 Hattori Botanical Laboratory, Obi 6-1-26, Nichinan, Miyazaki 889-2535, Japan Hattori Botanical Laboratory Nichinan Japan

**Keywords:** Asia, *Frullania*, gathering, lectotypificaion, liverworts, nomenclature, Oceania, *Porella*

## Abstract

Rediscovery and examination of the type specimen of *Frullaniaauriculata* S.Hatt. proves that the original type citation of this species contains an error, which is corrected here. Lectotypification for *Porellatakakii* S.Hatt. is provided.

## ﻿Introduction

*Frullaniaauriculata* S.Hatt., a rare liverwort endemic to Oceania, is known solely from its type collection (*N. Kitagawa 23455*; Hattori 1985; Pócs 2008; Söderström et al. 2011). While reorganizing the bryological collection housed in the Herbarium of the Hattori Botanical Laboratory (NICH 2024), only one specimen (*N. Kitagawa 23435*) was found, and its holotype (*N. Kitagawa 23455*) could not be located. Consequently, upon examination of this specimen, it was revealed that Hattori (1985) had utilized it, indicating potential inaccuracies in the citation of the original material in the protologue of *F.auriculata*, necessitating correction.

Moreover, in NICH, five collections of *Porellatakakii* collected from its type locality were identified, indicating the need for lectotypification of this species (Art. 9.3). To address these nomenclatural issues, the following resolutions are proposed:

## ﻿Taxonomy and typification

### ﻿1. Frullania auriculata

Taxon classificationPlantaePorellalesFrullaniaceae

S.Hatt., Bull. Natl. Sci. Mus. Tokyo, B 11: 11 (1985).

DA4A277E-64E3-5E31-BFF8-D30C24045076

#### Original material citation.

**Type.** Fiji. Mt. Victoria, 700–980 m alt. On tree trunk. 27 Aug. 1982. *N. Kitagawa 23435* (holotype: NICH 389176).

#### Note.

*Frullaniaauriculata* was described based on a specimen collected from Fiji (*N. Kitagawa 23455*; Hattori 1985). The protologue states, the holotype and isotype of *F.auriculata* were deposited in NICH and TNS, respectively. However, these types are not included in the catalogue of type specimens (Inoue 1987; Mizutani et al. 2009) or registered in the online databases of either herbarium (https://hattorilab.org/database/; https://type.kahaku.go.jp/TypeDB/search?cls=bryophyta).

**Figure 1.**
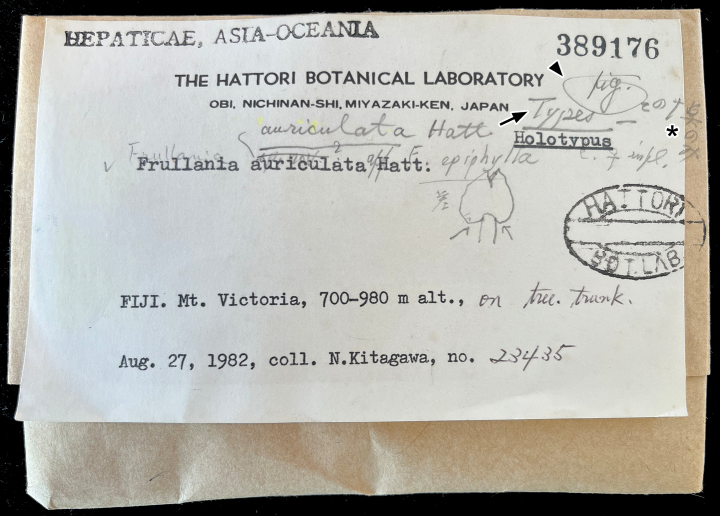
Specimen label of *N. Kitagawa 23435* (NICH 389176). Arrow, arrow head and asterisk indicate “Types”, “fig” and “この1点のみ” (= Only this one specimen), respectively, handwritten by Hattori.

Recently, I reorganized the bryological collection of NICH and found a suspected type of *Frullaniaauriculata* (*N. Kitagawa 23435*; Fig. 1). This specimen fits all the information provided in the protologue of *F.auriculata* (e.g., collection site, altitude, substrate, and collector) expect for the collecting number, which is “*23435*” rather than “*23455*” as in the protologue (Hattori 1985). However, several lines of evidence suggest “*N. Kitagawa 23435*” is the type of *F.auriculata*.

First, there are Hattori’s handwritten contents (species name, “type”, and “fig.”) on the label of “*N. Kitagawa 23435*”, which indicate that this specimen was examined, regarded as type, and used for drawing the illustration by Hattori himself (Fig. 1). Second, in the protologue of *F.auriculata*, Hattori (1985) cited only one specimen (*N. Kitagawa 23455*) and noted that this species was “known only from the type collection”. Hattori annotated the upper right corner of the specimen label “この1点のみ” (= Only this one specimen) (Fig. 1), which corresponds with the distributional statement provided in the protologue (Hattori 1985).

**Figure 2.**
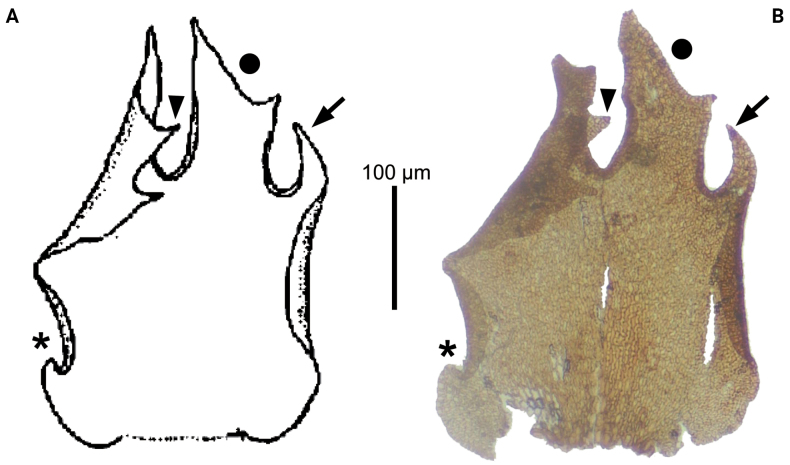
Innermost female bracteole of *Frullaniaauriculata* S.Hatt. **A** from the original illustration (Hattori 1985) **B** from *N. Kitagawa 23435* (NICH 389176). Arrows, arrow heads, asterisks and circles indicate the morphological similarities between A & B.

The innermost female bracteole of “*N. Kitagawa 23435*” perfectly matches the original illustration (Fig. 2). It should be noted that other parts of plants illustrated in the protologue were not located. These evidences seem to support that this newly located specimen (*N. Kitagawa 23435*) is the one used by Hattori himself to prepare the protologue of *F.auriculata* (Hattori 1985). Therefore, the type citation of *F.auriculata* should be corrected in accordance with Art. 9.2 of the International Code of Nomenclature (Turland et al. 2018), which is provided in the above section.

The misspelling of specimen numbers is not unique to *Frullaniaauriculata*, also occurring in the type citation for Frullaniaepiphyllasubsp.fijiensis S.Hatt., described in the same study as *F.auriculata* (Hattori 1985), see Zheng (2024).

### ﻿2. Porella takakii

Taxon classificationPlantaePorellalesPorellaceae

S.Hatt., J. Jap. Bot. 28: 181 (1953).

D934B4D2-CBA7-5624-8583-C51DF0787E31

≡ Porellaoblongifoliavar.takakii (S.Hatt.) Inoue, Bull. Chichibu Mus. Nat. Hist. 6: 28 (1955).

#### Original material citation.

**Type.** Japan. Nagano County, Miwa-mura, shaded damp limestone ledge, associated with *P.parvistipula*, *Quercuscrispula* woods in upper part of Shiroiwa valley, *ca.* 1300 m alt. July 1952. *N. Takaki s.n.* (lectotype: NICH 41848, in Hepaticae Japonicae Ser. 5, no. 241, here designated).

#### Note 1.

*Porellatakakii* was published by Hattori (1953), and is considered to be closely related to *P.oblongifolia* S.Hatt. but can be distinguished by its teeth-present leaves and leaf-lobules (Hattori 1943, 1953). Later, Inoue (1955) considered the characters variable and reduced *P.takakii* to a new variety of *P.oblongifolia*. Hara (1955) could not distinguish between the two species and synonymized *P.takakii* under *P.oblongifolia*, which was followed by Hattori (1978).

**Figure 3.**
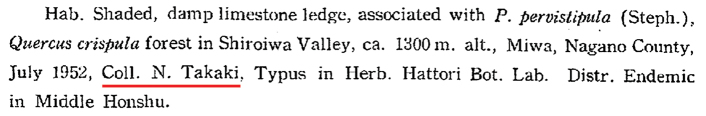
Type citation of *Porellatakakii* S.Hatt. in its protologue (Hattori 1953). Only substrate, ecological habitat, collection site, altitude, date and collector were given. The red underline indicates the absent specific number.

Although *Porellatakakii* has been repeatedly researched, its original citation remains unclear because no specimen number was provided (Hattori 1953; Fig. 3). I investigated the original materials of *P.takakii* deposited in NICH and found that nine preparations belonging to five gatherings fit the original locality: (1) *N. Takaki 327* (NICH 51476, 51477, 51478, 51479, and 51480), (2) *N. Takaki 328* (NICH 51534), (3) *N. Takaki s.n.* (NICH 20909), (4) *N. Takaki s.n.* (NICH 41848, in Hepaticae Japonicae Ser. 5, no. 241), and (5) *N. Takaki s.n.* (NICH 60513). The latter three specimens share an absent collection number, but they should be considered separate gatherings because they are neither cross-labelled nor bear a single and original label in common (Art. 8.3; Turland et al. 2018). However, none of these specimens perfectly align with the original ecological data (Fig. 3). For instance, “*N. Takaki 327*” (NICH 51476, 51477, 51479, and 51480) were collected from a “shaded, damp ledge of limestone”, while “*N. Takaki 327*” (NICH 51478) originated from a “*Quercuscrispula* forest, on shaded, damp ledge of limestone”. Similarly, “*N. Takaki 328*” (NICH 51534) was found in a “*Quercuscrispula* forest, shady limestone”, and “*N. Takaki s.n.*” (NICH 20909) from “associating with *P.grandiloba*, on shaded, damp ledge of limestone”; “*N. Takaki s.n.*” (NICH 60513) were collected from “on calcareous rock under *Quercus* woods”. Only “*N. Takaki s.n.*” (NICH 41848, in Hepaticae Japonicae Ser. 5, no. 241) precisely matches the original ecological citation (Hattori 1954). It can be thus inferred that exicattae set contains isosyntypes, implying equal prioritisation of lectotype selection with other specimens listed above according to the Art. 9. 12 (Turland et al. 2018). It is worth noting that Mizutani et al. (2009) regarded “*N. Takaki 328*” (NICH 51534) as the holotype and “*N. Takaki s.n.*” (NICH 20909) as the isotype of *P.takakii*; however, this was not the case. All five gatherings noted above were considered syntypes of *P.takakii* (Art. 9.6; Turland et al. 2018).

Due to the difficulty in assessing the condition of distributed exicattae specimens, only “*N. Takaki s.n*.” (NICH 41848) was examined, confirming its alignment with the original description and illustration, particularly in its apically toothed leaves, the most distinguishing characteristic of this species. Consequently, this specimen was designated as the lectotype of *P.takakii*, despite some plants displaying mechanical damage and lacking intact leaf apices (data not shown).

There seems to be an inconsistent issue that *Porellatakakii* was recorded as associating with “*P.parvistipula* (Steph.)” (Probably an orthographic variant of *P.parvistipula* (Steph.) S.Hatt.) in the protologue (Fig. 3) but occurring with *P.grandiloba* Lindb. (NICH 20909). Since the former species (*P.parvistipula*) has been listed as one of the synonyms of the latter (*P.grandiloba*), both statements are actually the same (Hattori 1978).

## Supplementary Material

XML Treatment for Frullania auriculata

XML Treatment for Porella takakii

## ﻿Additional information

### Author ORCIDs

Tian-Xiong Zheng https://orcid.org/0000-0002-1963-2302

### Data availability

All of the data that support the findings of this study are available in the main text.
